# Topical Lidocaine plus Diclofenac as a Local Anesthetic Agent in Central Venous Catheterization; a Randomized Controlled Clinical Trial

**DOI:** 10.22037/aaem.v9i1.1389

**Published:** 2021-09-15

**Authors:** Reza Azizkhani, Maysameh Shahnazari Sani, Farhad heydari, Mina Saber, Sarah Mousavi

**Affiliations:** 1 Department of Emergency Medicine, Faculty of Medicine, Isfahan University of Medical Sciences, Isfahan, Iran.; 2 Department of Dermatology, School of Medicine, Isfahan University of Medical Sciences, Isfahan, Iran.; 3 Department of Clinical Pharmacy and Pharmacy Practice, School of Pharmacy and Pharmaceutical Sciences, Isfahan University of Medical Sciences, Isfahan, Iran.

**Keywords:** Diclofenac, Anesthetics, Local, Lidocaine, Central Venous Catheters, Pain Management

## Abstract

**Introduction:**

Various methods of analgesia can be used to reduce or prevent procedural pain in emergency department (ED). This study aimed to evaluate the effectiveness of topical lidocaine-diclofenac combination compared to lidocaine-prilocaine combination (Xyla-P) in reduction of the pain during central venous catheter (CVC) insertion.

**Methods:**

In this randomized clinical trial, 100 adult patients requiring CVC insertion in the ED were enrolled. These patients were randomly divided into two groups. The site of CVC insertion was covered with 2 g of topical Xyla-P cream in the first group, and 2 g of topical lidocaine-diclofenac cream in the second group. The primary outcome was the pain during CVC implantation. The secondary outcomes were physician satisfaction and the incidence of side effects.

**Results:**

On the visual analog scale (VAS), the pain score during CVC insertion was significantly lower in the second group (p = 0.027). However, there was no difference in pain scores during lidocaine injection between the two groups (p = 0.386). Also, there was no significant difference in the rate of side effects between the two groups (p = 1.0). The physician’s satisfaction with the first group was significantly lower than the second group (p = 0.042).

**Conclusion:**

Although the CVC insertion pain was significantly lower in patients who received the topical combination of Lidocaine plus Diclofenac, there was no clinically important difference between the two groups and both topical anesthetics were effective and safe in reducing pain intensity. Also, lidocaine-diclofenac combination cream was more cost-effective than Xyla-P cream.

## 1. Introduction:

Central venous catheter (CVC) insertion is one of the most frequently performed invasive procedures in the emergency department (ED), which is associated with pain, anxiety, and discomfort (1, 2). The pain felt during the procedure is commonly reduced with the use of local anesthetics such as lidocaine. However, injection of local anesthetics itself may be associated with pain at the site of injection (1, 3).

Most clinicians believe that the local anesthetic injection will cause more pain than subsequent procedures, such as anchoring the catheter to the skin with sutures or eventually threading the dilator over the guidewire (4). Pain during catheterization can cause anxiety and may negatively affect the treatment received. Ensuring patient comfort is also important for increasing cooperation and contributing to procedure facilitation, thus reducing the risk of insertion failure or catheter malpositioning (5).

Various methods of analgesia can be used to reduce or prevent procedural pain. Using topical anesthetics is an available, low-cost, and effective method to achieve analgesia. Topical drug administration has clear advantages over other routes of administration, such as high levels of efficacy, more safety, and patient compliance. There is good evidence that adults benefit from reducing discomfort and anxiety by using an effective topical analgesic cream at the site of the procedure (6-9).

For optimal pain management, EDs have a vast variety of protocols for reducing pain. Local anesthesia with Xyla-P cream 5% (2.5% lidocaine and 2.5% prilocaine) has been shown to effectively reduce pain associated with minor procedures such as needle punctures (7, 9). Another option is a nonsteroidal anti-inflammatory drug (NSAID), such as transdermal diclofenac patch (TDP), which is available and effective in reducing chronic pain by reducing prostaglandin synthesis (10-12).

Both lidocaine and diclofenac have analgesic, anti-inflammatory, and antibiotic effects (10-13). Also, lidocaine and diclofenac have a synergistic analgesic effect (14, 15). The dual mechanism of action of these substances on the cellular level is functionally synergistic in pain control. The combination of these agents results in a more long-lasting analgesia than that obtained with any of the substances alone (14). The pathophysiological mechanisms of pain are complex; therefore, combining active drugs with multiple mechanisms and synergistic action is a potentially more effective therapeutic approach to pain management than conventional monotherapy (6). This study aimed to evaluate the effectiveness of topical lidocaine-diclofenac combination compared to the Xyla-P cream in reduction of the pain during CVC insertion.

## 2. Methods

*2.1. Study design and setting*

This prospective randomized double-blind clinical trial was conducted in the ED of two university teaching hospitals (Alzahra and Kashani Hospitals) in Isfahan, Iran, from January 2020 to April 2021. The study was approved by the Ethics Committee of Isfahan University of Medical Sciences (IR.MUI.MED.REC.1398.063). The trial was registered on the Iranian Registry of Clinical Trials under the number IRCT20180129038549N11. All patients provided written informed consent for participation in the trial.

*2.2. Participants*

All adult patients requiring CVC insertion in the ED consented to participating and were enrolled in the study. Patients were included in the study if they were older than 18 years, awake, alert, and oriented, and their medical condition was stable enough to allow CVC to be inserted within about 1 hour. Patients with visual, mental, or verbal disorders, a history of an allergic reaction to local anesthetics, a history of favism, methemoglobin, renal and liver disease, skin diseases at or around the CVC insertion site, and a history of drug addiction, a history of analgesic use within 24 hours before the procedure, were excluded. Also, patients were excluded if the venous catheter placement was not successful the first time (skin puncture was repeated more than once). 

*2.3. Intervention*

The patients were randomly allocated to receive one of the two topical anesthetics: Xyla-P cream (lidocaine 2.5% and prilocaine 2.5%), or lidocaine-diclofenac cream (2% lidocaine and 1% diclofenac). Randomization was based on a random-allocation software package (1:1).

In the first group (LP group), the site of CVC insertion was covered with 2 gr of Xyla-P cream (Tehran Chemie Pharmaceutical Company, Iran). In the second group (LD group), the site of CVC insertion was covered with 2 g of a fixed-dose combination containing 1% diclofenac (Sobhan Darou Company, Iran) and 2% lidocaine (Sina Darou Company, Iran) cream. The topical anesthetic was applied on a 5 cm ^2^ surface area over the procedure site in a thick layer and covered with an occlusive dressing for at least 45 min before the CVC implantation.

Lidocaine-Diclofenac cream was prepared in 30 g weighted tubes in collaboration with the Faculty of Pharmacology (Isfahan University of Medical Sciences). It was matched with the Xyla-P cream in terms of color, smell, and shape, as well as labeling. An independent investigator who was not involved in clinical management and data collection did the randomization and prepared topical creams every day. 

After 45 minutes (16), the dressing and cream were removed. The blinded investigator then injected 5 ml of 2% lidocaine through a 25-gauge needle. The investigator injected 3 mL of lidocaine directly superficial to the internal jugular vein, then injected 1 mL just to the left and 1 mL just to the right of the vein for anchoring stitches. Five minutes after injection, an attempt was made to insert CVC into the right internal jugular vein using the anterior approach with ultrasound guidance. Each patient received a 7 Fr triple-lumen catheter via a non-tunneled approach. 

*2.4. Data gathering*

All measurements were recorded by investigators blinded to randomization and the type of topical analgesia used. The pain was assessed using a Visual Analog Scale (VAS) from 0 to 10 (0: No pain, 10: The worst possible pain imaginable) (6, 7). The physician’s satisfaction was assessed using a 10-point verbal numeric rating scale from 0 to 10 (0: Completely dissatisfied, 10: Completely satisfied).

The pain scores were reported by the patient after initial subcutaneous lidocaine injection, and just after CVC insertion. The physician’s satisfaction was recorded after the overall procedure was completed. In the beginning of the study, patient characteristics (age, sex, and body mass index (BMI)) were recorded. During the study, an investigator evaluated the local side effects (erythema, urticaria, pruritus, and irritation). Systemic effects (heart rate and systolic and diastolic blood pressure) were also recorded before intervention and after CVC insertion. The patients, physicians, and nurses who participated in the trial were blinded to the randomization.

*2.5. Outcomes*

The primary outcome was the intensity of pain during CVC implantation. The secondary outcomes were physician satisfaction and the incidence of side effects.

*2.6. Statistical Analysis*

Calculations showed that to detect a standard deviation of 0.90 for pain scores and a difference of 0.54 (17) on the VAS scale (score range from 1 to 100) during CVC insertion with 95% confidence interval, and a power of 80%, a sample size of 45 patients was required in each group. Thus, the study population of 50 patients per group was considered based on an anticipated dropout rate of 10% to ensure an adequately powered study.

Finally, the collected data was analyzed using SPSS software (ver. 25) and they were shown as Mean ± standard deviation (SD) or frequency (%). Chi-square test was used to compare qualitative data between the two groups, independent t-test and paired t-test were used to compare the mean of quantitative data, and univariate analysis was used to compare the mean pain score by adjusting confounding factors, such as age, sex, and BMI. The level of significance was considered less than 0.05.

## 3. Results:

**Figure 1:**
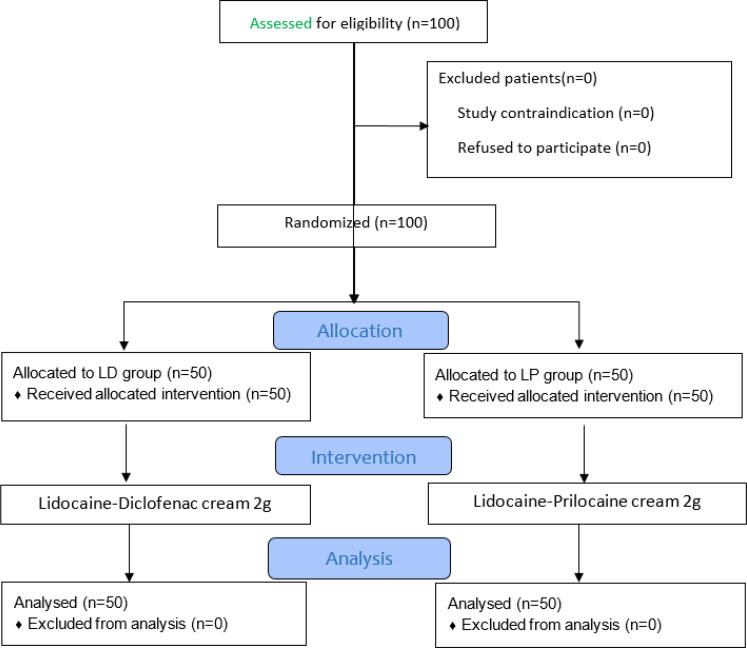
CONSORT flow diagram of the study.

A total of 100 patients (50 patients per group) were enrolled in this study. All patients were included in the analysis (Figure 1). The mean age was 36.15 ± 7.36 years (range 18–59); 61.0% were male. There was no statistically significant difference in baseline characteristics between the two groups (Table 1).

The mean pain scores during lidocaine injection and CVC insertion are reported in Table 2. The pain scores during CVC insertion were significantly lower in the LD group compared to the LP group (3.74 ± 2.14 vs 4.60 ± 2.04, P = 0.027), however, there was no difference in pain scores during lidocaine injection between LD and LP groups (P = 0.386). 

The physician’s satisfaction in the LD group was higher than the LP group (P = 0.042). There was no episode of urticaria and edema at the site of application in either of the groups. There was no significant difference in the rate of side effects between the two groups (Table 2). Also, there was no significant difference in vital signs (systolic blood pressure, diastolic blood pressure, and heart rate) between the LD and LP groups before and after the CVC insertion (p > 0.05) (Table 3).

## 4. Discussion:

According to the results of the present study, although the CVC insertion pain was significantly lower in patients who received the topical combination of Lidocaine plus Diclofenac, there was no clinically important difference between the two groups and both topical anesthetics were effective and safe in reducing pain intensity. Also, lidocaine-diclofenac combination cream is more cost-effective than Xyla-P cream.

Procedural pain relief or control not only reduces anxiety and fear in patients but also increases their cooperation and contributes to the ease of the procedure and improves overall patient satisfaction. A CVC insertion can cause much pain and anxiety. One way to reduce this pain and anxiety is to use topical and local anesthesia.

Linares-Gil et al. (2018) showed that a topical formulation containing lidocaine plus diclofenac was safe and more effective than the topical lidocaine alone for reducing the pain intensity during the first three days after surgery in benign anorectal surgery (15). 

Topical NSAIDs are effective in decreasing acute and chronic pain by inhibiting prostaglandin synthesis at the site of application (18). Khalili et al. (2014) demonstrated that the diclofenac gel significantly reduced the pain severity associated with vein catheter insertion and was more effective than EMLA (containing Lidocaine/prilocaine) (17). They suggested using diclofenac gel rather than EMLA because it was more costeffective and effective in reduction of pain with fewer side effects.

Babaieasl et al. (2019) compared the efficacy of EMLA and Topical Diclofenac Patch (TDP) in attenuating peripheral venous catheters pain and phlebitis. EMLA and TDP had similar analgesic effects, but the incidence of phlebitis in the TDP group was significantly lower than the EMLA group (19). Contrary to other studies, Deshpande et al. (2010) showed higher effectiveness of EMLA cream in comparison to Diclofenac Transdermal Patch for attenuation of the pain caused by IV cannulation among adult patients (18). These studies/This study, similar to the present study, demonstrated the efficacy of topical diclofenac in reducing pain during venous cannulation.

The pain caused by lidocaine injections is often considered a necessary problem, but there are several ways to reduce it. Buffering and warming of the lidocaine solution before injection are probably the simplest and most effective measures (20, 21). Another measure to reduce injection pain is to use topical anesthesia. In the present study, the application of both creams was effective in decreasing the pain of lidocaine injection. It should be noted that lidocaine injections as deep as 2–3 cm in the neck were performed in the current study, in contrast to intradermal and immediate subdermal injections most often received in the hand or arm, as in most other similar studies.

Culp et al. (2008) compared various local analgesics regarding their efficacy of pain reduction during central venous catheter placement. There was no difference in pain scores during lidocaine injection between lidocaine and buffered lidocaine groups. Contrary to the present study, they showed that the mean score of pain during lidocaine injection was higher than pain during the insertion of the catheter (22). But in the present study, mean pain score during lidocaine injection was lower than CVC insertion in both groups. Since there was no control group in this study, we could not have evaluated the pain of CVC insertion in patients who only received lidocaine injection without any topical analgesia.

Selvi et al. demonstrated that topical vapocoolant spray can be used before digital nerve blocking to reduce procedural pain (23). Heydari et al. showed that local cutaneous ketamine is as effective as EMLA for reducing the pain during venipuncture (7).

There was no episode of urticaria or edema at the site of application in either of the groups. Both erythema and irritation were observed in 2.0% of the participants in each group. There was no significant difference in the rate of side effects between the two groups. Similarly, Agarwal A et al. (2006) demonstrated no incidence of blanching at the site of DTP patch (10). In other articles, the most common side effect was blanching in the DTP and EMLA groups. These results showed that the use of topical diclofenac (patch or gel) before intravenous cannulation can be an effective and safe way to reduce pain (17, 18).

**Table 1:** Comparing the baseline characteristics of patients between two groups

**Variables**	**LP group (n=50)**	**LD group (n=50)**	**P value**
**Sex**			
Male	33 (66.0)	28 (56.0)	0.305
Female	17 (34.0)	22 (44.0)	
**Age (year)**	36.84 ± 7.60	35.45 ± 7.37	0.084
**BMI (kg/m** ^2^ **)**	28.50 ± 2.51	28.32 ± 2.36	0.713

Data are presented as mean ± standard deviation or frequency (%). LP: Lidocaine-Prilocaine; LD: Lidocaine-Diclofenac; BMI: body mass index.

**Table 2:** Comparison of pain scores, physician satisfaction, and side effects between the two groups

**Variables**	**LP group (n=50)**	**LD group (n=50)**	**P value**
**Pain (based on VAS)**			
During lidocaine injection	1.68 ± 0.41	1.62 ± 0.47	0.386
During CVC insertion	4.60 ± 2.04	3.74 ± 2.14	0.027
**Physician satisfaction**			
Mean ± SD	6.40 ± 2.040	7.26 ± 2.136	0.042
**Side effects **			
Erythema	1 (2.0)	1 (2.0)	NA
Irritation	1 (2.0)	1 (2.0)	

Data are presented as mean ± standard deviation (SD) or frequency (%). LP: Lidocaine-Prilocaine; LD: Lidocaine-Diclofenac; CVC: central venous catheter; VAS: visual analogue scale.

**Table 3:** Comparison of vital signs before and after central venous catheter insertion in the two groups

**Variables**	**Vital Signs**		**P value**
	**Before Intervention**	**After Catheterization**	
**Systolic Blood Pressure (mmHg)**			
LP group	125.43 ± 15.78	122.69 ± 15.71	0.383
LD group	123.38 ± 14.73	124.87 ± 15.56	0.465
**Diastolic Blood Pressure (mmHg)**			
LP group	79.71 ± 9.95	81.45 ± 10.13	0.214
LD group	80.35 ± 10.65	82.65 ± 10.11	0.578
**Heart Rate (beat /minute)**			
LP group	82.88 ± 11.65	85.58 ± 12.23	0.245
LD group	82.61 ± 11.09	84.64 ± 11.72	0.212

Data are presented as mean ± standard deviation. LP: Lidocaine-Prilocaine; LD: Lidocaine-Diclofenac.

## 5. Limitations:

Small sample size and evaluation of pain severity via a subjective method (VAS) can be considered as limitations of the present study. There was no control/placebo group for the comparison of VAS score to lidocaine injection. Minimum duration of 45 minutes was allowed for the application of both creams, which is a rather long period in the ED, as most of the studies using EMLA and NSAIDs for topical application have shown that 45-60 minutes is required for their full effect. The skin thickness affects topical absorption of the drug, so further studies can assess this matter more accurately. It is also suggested to do future studies to evaluate the effect of the present drug combination at different times and in different procedures to generalize the results of the present study to the community with more certainty.

## 6. Conclusion:

According to the results of the present study, although the CVC insertion pain was significantly lower in patients who received the topical combination of Lidocaine plus Diclofenac, there was no clinically important difference between the two groups and both topical anesthetics were effective and safe in reducing pain intensity. Also, lidocaine-diclofenac combination cream is more cost-effective than Xyla-P cream.

## 7. Declarations:

### 7.1. Acknowledgments

The present article was extracted from the thesis by Dr. Maysameh Shahnazari Sani to achieve her specialist degree in emergency medicine from Isfahan University of Medical Sciences. The authors would like to express their gratitude to the staff of the ED of Al-Zahra and Kashani Hospitals, Isfahan, Iran. 

### 7.2. Authors' Contributions

R.A., M.S.S., F.H., S.M., and M.S.; Contributed to conception, study design, and data collection and evaluation. R.A., M.S.S., F.H.; Contributed to statistical analysis, and interpretation of data. R.A., F.H.; Were responsible for overall supervision. F.H. and M.S.S; Drafted the manuscript, which was revised by R.A., M.S. and S.M. All authors edited and approved the final version of this paper for submission, and also participated in the finalization of the manuscript and approved the final draft.

### 7.3. Conflict of Interest

The authors declare no conflict of interest. 

### 7.4. Funding

This study was funded by Isfahan University of Medical Sciences.
